# Shifting to Primary Prevention for an Aging Population: A Scoping Review of Health Promotion Initiatives for Community-Dwelling Older Adults in Canada

**DOI:** 10.7759/cureus.17109

**Published:** 2021-08-11

**Authors:** Christina Visconti, Elena Neiterman

**Affiliations:** 1 School of Public Health and Health Systems, University of Waterloo, Waterloo, CAN

**Keywords:** gerontology, health promotion, canada, health policy, community health services

## Abstract

Older adults are healthier and living independently within our communities for longer. This demonstrates the need to build capacity in geriatric preventative services, with the potential to utilize health promotion to encourage successful aging. This scoping review examines the availability and potential of health promotion initiatives for community-dwelling older adults in Canada. Two research databases were searched for empirical articles published in Canada, in English, since 2000. A total of 17 articles met our inclusion criteria. The empirical literature demonstrates successful implementation of different primary prevention programs, with various facilitation methods used to address several health issues in late life. Most programs targeted falls prevention, often using education or exercise programming. Participants reported positive results in various biopsychosocial aspects of aging. Reported positive health outcomes and high engagement rates across examined programs may represent the ability for health promotion to successfully address the needs of older adults in the community, as well as meet the existing desire for participation in such initiatives. Further implementation and investment into health promotion for older adults can increase the accessibility of these programs across Canada, address new needs amongst this population, and alleviate the future healthcare burden posed by the growing aging demographic. The need for preventative services in gerontology is universal, thus the success seen in health promotion programs and policy, and the need for expansion, in Canada may also be relevant in countries with similar demographics.

## Introduction and background

Population aging is a growing global phenomenon [1]. In 2020, 18% of the Canadian population, or about 6.8 million individuals, were over the age of 65 years [2], with approximately 93% of Canadian older persons residing in private dwellings [3]. By 2030, 23% of the population will be older adults, as life expectancy in Canada is predicted to increase from 84.2 years to 86.2 years [3]. With Canadians staying healthier and living in the community for longer, solutions to address the needs and mitigate the burden of the aging population are essential [4].

Rowe and Khan’s concept of “successful aging” defines three goals for optimal aging: (1) minimal disease and disability, (2) high cognitive and physical functioning, and (3) meaningful engagement in life [4]. This definition emphasizes biopsychosocial contributors to healthy aging through establishing the importance of preventing decline and empowering the individual, in addition to disease management, in later life [5]. Overall, older adults hope to successfully age by maintaining their independence and health [6]. Preventative services provide the potential for improved physical function, increased social engagement among older adults, and consequent healthcare cost reduction [7].

Public health initiatives are classified by three levels of prevention [8]. Primary prevention looks to prevent disease before it begins, reducing the overall incidence of a disorder [8]. Secondary prevention aims to detect disease in its early stages to limit its prevalence [8]. Lastly, tertiary prevention focuses on rehabilitation and limiting severe impairment from an existing disorder [8]. With older adults maintaining their health for longer, focusing on primary prevention can enable individuals to successfully age while also limiting the burden of disease on the healthcare systems. To better empower, educate, and prevent illness for aging populations around the world, health promotion presents a promising outlet.

According to the World Health Organization, health promotion is the process of enabling individuals to take control over and improve their health [9]. Effective health promotion contributes to more efficient health service usage, decreased morbidity and disability, and overall higher life expectancy and quality of life [10]. Community health promotion programs, along with health education by primary care providers, can support successful aging and primary prevention through encouraging self-management and healthy living behaviors in later life. These behaviors can prevent comorbidities, increase one’s ability to manage change, and enable older adults to continue being contributing members of society [6].

The benefits of health promotion programming can translate into successful aging, but to confirm its value for implementation, it is important to review evidence on the experiences of participants and characteristics of program facilitation for existing initiatives. This will enable evidence-based decisions in public health policy on where to allocate resources and how to further utilize health promotion as a useful tool for the older population in Canada. This scoping review aims to achieve this by summarizing the empirical literature on the availability of health promotion programs for community-dwelling older adults in Canada. No knowledge synthesis in this area of health promotion and gerontology exists with a focus on preventative programs, as the large majority of literature on this topic focuses solely on disease management. Therefore, the following research questions are posed: what health promotion initiatives for community-dwelling older adults in Canada have been described in the empirical literature? What are the types and foci of these initiatives? What recommendation(s) can be drawn from the literature on how to effectively implement health promotion initiatives for older adults? 

The growing literature on health promotion and the need for evidence-based interventions to support community-dwelling older adults in Canada exemplify the timeliness and necessity for this review.

## Review

A scoping review was the best tool to broadly, but systematically, map the empirical literature on this topic.

Methods

Arksey and O’Malley’s five-stage methodological framework for conducting a scoping review was utilized for this synthesis and narrative analysis [11]. The review was conducted in consultation with the University of Waterloo librarian on search terms and approaches.

Stage 1: Identifying the Research Question

A broad research question was developed to maintain the breadth of coverage of the literature. The main research question was: “what health promotion initiatives for community-dwelling older adults in Canada have been described in the empirical literature?” The following sub-questions were also posed: what are the types and foci of these initiatives? What recommendation(s) can be drawn from the literature on how to effectively implement health promotion initiatives for older adults?

Stage 2: Identifying Relevant Studies

Inclusion criteria were determined based on a preliminary scan of the literature. Empirical studies published in English in the past 20 years (January 2000 to October 2020) and conducted in Canada were included in the review. Implemented initiatives with health promotion, health education, or a primary prevention focus were considered part of the inclusion criteria. Older adults were defined as individuals 60 years and older to narrow a population sample. Lastly, “community-dwelling” individuals were defined as those who live in private dwellings or in community settings. Programs targeting older adults residing in nursing, retirement, or long-term care homes and/or hospitals were excluded from the review. Non-systematic reviews and non-empirical articles (guidelines, opinions, frameworks) were also excluded from this review.

Searches were conducted on the PubMed and CINHAL electronic databases after determining this best aligned with the subject-area of the review. In consultation with the librarian, it was agreed upon that searches of medical subject heading (MeSH) and title/abstract would be best to avoid noise and retrieve the most accurate results. Search terms used included: (health promotion[MeSH] OR health promotion[tiab] OR health education[MeSH] OR health education[tiab]), (older adult*[tiab] OR seniors[tiab] OR elders[tiab] OR elderly[tiab] OR aged[mesh] OR old age[tiab]), (community participation[MeSH] OR community[tiab] OR community-based[tiab] OR home[tiab] OR home-based[tiab]), and (Canada[tiab] OR Canadian[tiab] OR Canada[MeSH] OR Newfoundland[tiab] OR Labrador[tiab] OR Prince Edward Island[tiab] OR Nova Scotia[tiab] OR New Brunswick[tiab] OR Quebec[tiab] OR Ontario[tiab] OR Manitoba[tiab] OR Saskatchewan[tiab] OR Alberta[tiab] OR British Columbia[tiab] OR Northwest Territories[tiab] OR Yukon[tiab] OR Nunavut[tiab]). The initial search of academic literature produced 654 articles.

Stage 3: Study Selection

**Figure 1:**
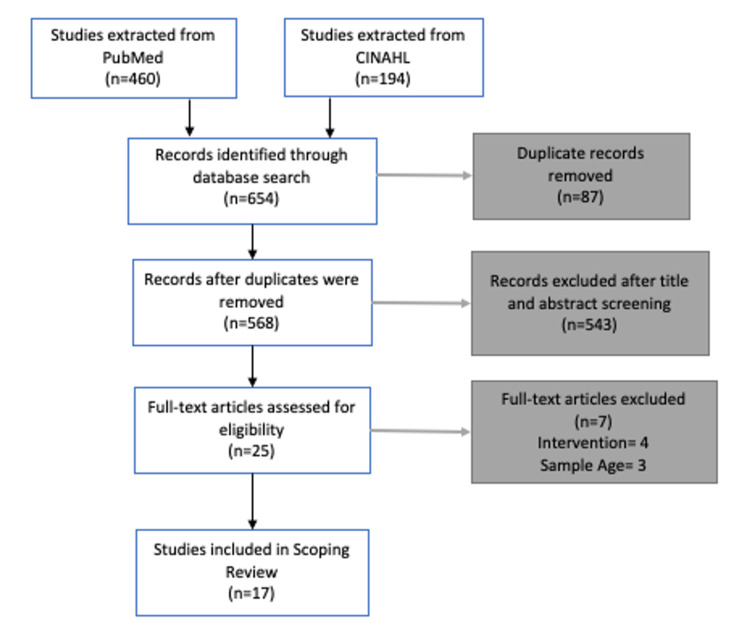
PRISMA Diagram for the Screening and Exclusion Process PRISMA: Preferred Reporting Items for Systematic Reviews and Meta-Analyses

A systematic process was used to select the literature included in this scoping review (Figure 1). First, results from each database search were imported into reference management software. Out of 654 results, there were 87 duplicates removed. Next, title and abstract screening was conducted on the remaining 568 studies. In total, 543 papers were determined out of scope and excluded from the review. Finally, 25 articles were assessed for eligibility through a full-text review, of which seven did not meet the inclusion criteria. Overall, 17 studies were included for charting and data extraction. 

Stage 4: Charting the Data

The data were charted using a literature extraction tool in Microsoft Excel. The categories for the extraction modeled goals of the research questions: author(s), date of publication, location (in Canada), study objectives, research methods used (qualitative, quantitative, or mixed-method), age of sample population, program description, intervention type (education, screening, exercise), location of initiative (community or residential setting), intervention facilitator, targeted health concern, key findings, and future recommendations. Themes were inductively developed after re-reading the literature, extrapolating pertinent connections between studies. Themes of senior engagement, empowerment and maintaining independence, and gender differences were agreed upon among team members and included in the literature extraction tool to further chart the data.

Stage 5: Collating, Summarizing, and Reporting the Results

Extracted data were aggregated into qualitative thematic summaries. To address the research questions, themes related to programming type and participant experiences with the initiatives were analyzed to structure this review. All findings were presented with the interest of knowledge users and policy professionals in mind, further adding recommendations and highlighting some benefits and challenges for program planning in this area of public health. We did not appraise included literature for quality, as specified for scoping reviews [11].

Review results

Study Characteristics

The final sample of collected articles included 17 papers (Table 1). The summary of results was done based on various intervention characteristics and participant themes. The majority of studies utilized qualitative methodological design (n=12), followed by quantitative studies (n=3) and mixed-methods (n=2). Most studies were conducted in Ontario (n=8), followed by Quebec (n=5), British Columbia (n=3), and Alberta (n=1). Notably, there were no empirical studies from the Atlantic provinces or Northern Canada. The program availability seen in this sample aligns with the population distribution in Canada but also reflects the unequal access to health promotion programs for individuals across the country.

**Table 1:** Characteristics of Reviewed Studies QC: Quebec; ON: Ontario; BC: British Columbia; AB: Alberta

Authors	Year	Province	Study Design	Sample Size	Age of Sample	Intervention Type	Intervention Setting	Intervention Facilitators	Target Health Concern
Botner [12]	2018	QC	Qualitative	116	60+	Education	Residential	Community Volunteers	Social Isolation
Bouchard et al. [13]	2013	QC	Mixed	25	65+	Education	Residential	Self-Implemented	Inactivity
Gleberzon [14]	2001	ON	Qualitative	16	Not Specified	Education	Community	Chiropractic Interns	Bone Health
Holliday et al. [15]	2015	BC	Qualitative	27	65+	Education	Community	Community Volunteers	Hearing Health
Laforest et al. [16]	2017	QC	Qualitative	294	60+	Education	Community	Community Volunteers	Cognitive Health
Tan et al. [17]	2004	ON	Qualitative	126	60+	Education	Community	Researchers	Burns
Markle-Reid et al. [18]	2006	ON	Quantitative	288	75+	Education	Residential	Nurses	General Maintenance of Health
Cowan et al. [19]	2009	ON	Mixed	460	62-90	Exercise	Community	Geriatricians	Inactivity
Brouwer et al. [20]	2003	ON	Qualitative	38	67 to 87	Education; Exercise	Community	Physiotherapist	Falls
Filiatrault et al. [21]	2007	QC	Qualitative	98	60+	Education; Exercise	Community; Residential	Community Volunteers	Falls
Robitaille et al. [22]	2005	QC	Qualitative	200	60+	Education; Exercise	Residential	Physiotherapist	Falls
Taing and McKay [23]	2017	ON	Qualitative	415	65+	Education; Exercise	Community	Fitness Instructors	Falls
McKay et al. [24]	2018	BC	Qualitative	458	60+	Education; Exercise	Community	Fitness Instructors	Inactivity
Agarwal et al. [25]	2015	ON	Qualitative	79	65+	Education; Screening	Residential	Multi-disciplinary team	Cardiovascular Disease
Kaczorowski et al. [26]	2008	ON	Quantitative	39 communities	65+	Education; Screening	Community	Multi-disciplinary team	Cardiovascular Disease
Johnson et al. [27]	2018	AB	Quantitative	134	60+	Exercise; Nutrition	Residential	Multi-disciplinary team	Falls
Moody and Phinney [28]	2012	BC	Qualitative	20	65+	Arts-based Activity	Community	Community Volunteers	Social Isolation

Intervention Characteristics

**Figure 2:**
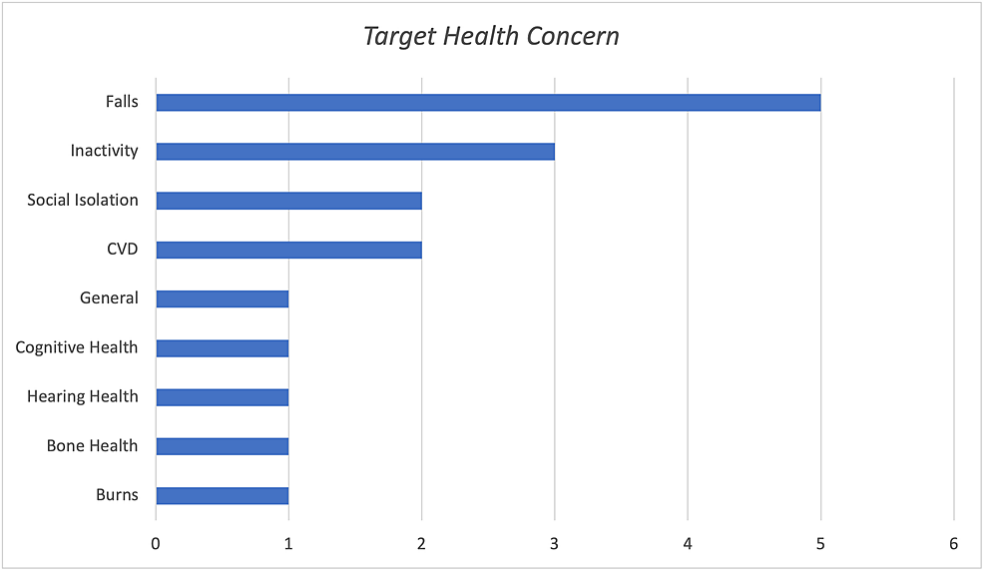
Target Health Concern of Programs Reviewed CVD: cardiovascular disease

The health promotion initiatives identified in this review were found to target different health concerns older adults may face (Table 1; Figure 2). Most commonly, the programs targeted falls (n=5), followed by physical inactivity (n=3), cardiovascular disease (CVD) prevention (n=2), social isolation (n=2), burns prevention (n=1), bone health (n=1), hearing health (n=1), cognitive health (n=1), nutrition (n=1), and general wellness (n=1) (Figure 2). Overall, the diversity within this small sample demonstrates the vast applicability of health promotion for the prevention of many concerns in later life.

**Figure 3:**
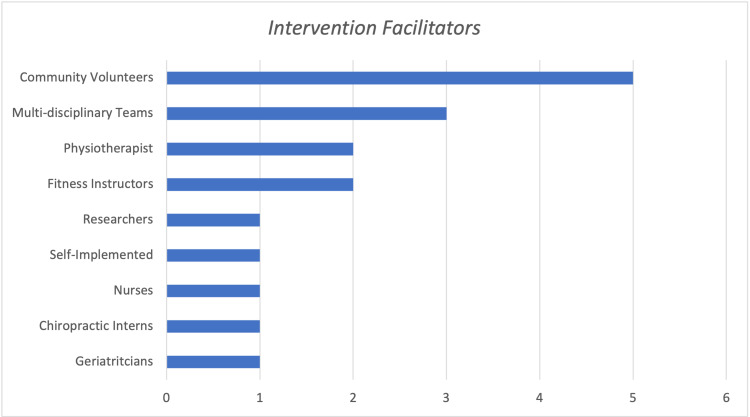
Facilitators of Study Interventions

The study sample demonstrated the multi-disciplinary nature of health promotion, as a variety of different stakeholders facilitated interventions (Figure 3). Most interventions were led by community volunteers (n=5) from regional facilities, followed by programs run by multi-disciplinary healthcare teams (n=3), physiotherapists (n=2), fitness instructors (n=2), chiropractic interns (n=1), nurses (n=1), geriatricians (n=1), researchers (n=1), and self-implemented by the participants (n=1).

**Figure 4:**
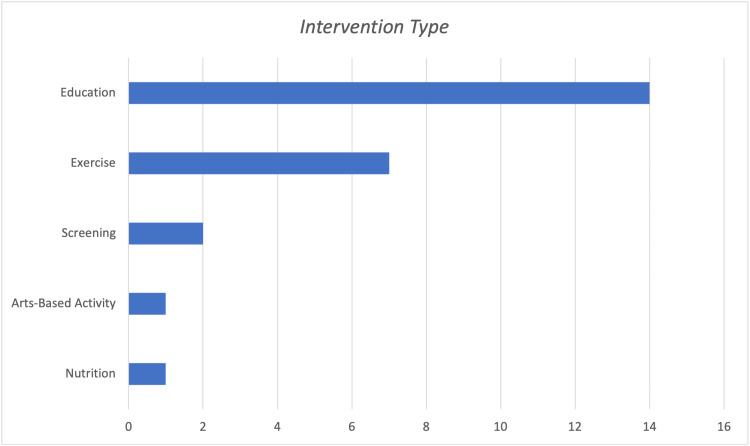
Intervention by Type

There were six demonstrated intervention types noted within the reviewed literature. Most interventions focused on educating older adults about the prevention of health problems, while others included hands-on components. Examples of practical approaches involved facilitating exercise classes, screening for disease onset factors, and providing dietary plans through nutrition guidance. Within this sample, an education approach was utilized most prevalently (n=14), followed by exercise (n=7), screening (n=2), nutrition (n=1), and an arts-based activity (n=1) (Figure 4). Many studies used a combined approach, i.e., education and exercise (n=5), education and screening (n=2), and education and nutrition (n=1) were often paired.

The following describes how these six different types of interventions were utilized with health promotion methodology for older adults in the reviewed studies.

Education: Seven studies used education for program facilitation, targeting health issues including social isolation [12], inactivity [13], bone health [14], hearing health [15], cognitive health [16], burns prevention [17], and general health [18]. The focus of these initiatives was to increase knowledge of the issue and share prevention tools. Educational sessions were delivered as either weekly workshops [12,16,18] or single-time presentations [13-15,17]. Botner implemented weekly sessions engaging participants through digital media, like videos and webcasts, to increase opportunities for technical learning and improved mental health. Despite difficulty in recruiting participants, enrolled older adults were highly satisfied with the program, reporting increased community connectedness [12]. Bouchard et al. facilitated a single educational session on the World Health Organization’s physical activity guidelines for older adults, enabling participants to choose a tracking tool (pedometer, manual pulse tracking, or heart rate monitor) to better identify their own activity and intensity levels. Results demonstrated that knowledge of tools and physical activity guidelines allowed participants in all groups to correctly identify recommended exercise intensity, although not to a statistically significant level [13]. 

Exercise: Given the large sample of initiatives focused on physical health, exercise was frequently utilized for primary prevention of falls and general health promotion. The majority of studies reviewed (n=7) combined exercise with other facilitation methods. Only Cowan et al. utilized solely exercise for the physical maintenance of frail community-dwelling older adults in Ontario. Drop-in exercise sessions were led by geriatricians at a regional community center [19]. After one year, findings indicated a decrease in health service use and increase in grip strength, but no change in health-related quality of life [19]. 

Education and exercise: Education and exercise were most commonly combined in the review, focused only on physical health, specifically falls [20-23] and physical inactivity [24]. The interventions were implemented in the same style, consisting of group exercise classes and discussion sessions for participants either before physical activity [24] or scheduled sporadically with it [20-23]. Participants in these programs reported decreases in mobility limitations [24] and increases in balance and strength [20-23]. Improved self-confidence in one’s abilities [20,23] and social inclusion were cited as additional benefits of participating in these initiatives [24]. Overall, using both group exercise and educational workshops aided biopsychosocial factors surrounding physical health [22]. 

Education and screening: Two interventions incorporating education and screening aimed at preventing the onset of cardiovascular disease. Kaczorowski et al. and Agarwal et al. implemented the Community Health Assessment Program (CHAP) in Ontario [25,26]. Kaczorowski et al. held the program within pharmacies, inviting participants to cardiovascular risk assessment sessions where blood pressure measurements were taken, along with discussions on how to reduce modifiable risk factors [26]. Agarwal et al. assessed the feasibility of the same initiative facilitated by emergency medical services (EMS) in a subsidized housing complex [25]. Overall, CHAP demonstrated positive impacts on blood pressure and lifestyle factors associated with chronic disease, and the ability to utilize low-resource health professionals for health promotion [25,26]. 

Nutrition and exercise:Johnson et al. was the only study reviewed that tested a multifactorial nutrition program for falls prevention in rural Alberta. It compared the efficacy of a combined nutrition and exercise program to standalone exercise, nutrition, and control groups [27]. A physiotherapist led a home exercise program in both the exercise and nutrition-exercise groups [27]. The nutrition and nutrition-exercise groups received nutritional supplements, along with guidance from a dietitian [27]. The study concluded that the exercise group outperformed the others in improving functional capacity and overall well-being [27]. Unfortunately, the nutrition component was not statistically significant without the exercise component [27]. Although the multifactorial component of this program was marginally significant, this study demonstrated the feasibility of at-home initiatives for rural populations to promote aging-in-place successfully [27]. 

Other activity-based programs: Moody and Phinney implemented a social isolation intervention utilizing an art-based community engagement project. Older persons at risk for social exclusion attended workshops at a senior's center [28]. Led by a local artist, participants were encouraged to create an art piece each week [28]. At the end of the study, the program held an art gallery for participants to display their art pieces to the community, their family, and friends [28]. Results indicated that older adults felt a greater connection to their community through the relationships built with program peers and beyond [28]. This art-based community engagement activity proved successful at promoting social inclusion and decreasing social isolation, demonstrating the diverse methods of program facilitation that can be utilized in health promotion for older adults within the community [28].

Participant Characteristics

Senior engagement: Overall, strong participant engagement and adherence were demonstrated in the majority of initiatives reviewed (n=11). Program adherence was high [20], with studies boasting 75% [14,23], 78% [22], 85% [18], 90% [16], and 100% participation rates [28]. Older adults expressed their enthusiasm in stating the importance and need for these preventative initiatives [16], demonstrating high engagement rates with the materials, activities, and their program peers [12,16,24]. While older adults reported high satisfaction rates and strong participation adherence, recruitment of participants proved to be a challenge in at least one reviewed paper [12]. The authors attributed recruitment challenges to the mode of the program, set as a virtual learning initiative due to technical limitations it posed for the participants [12].

Empowerment and maintaining independence: Empowerment and independence were reported in a few (n=4) reviewed studies to characterize post-intervention effects on older adults. In feedback surveys of the programs studied, participants expressed that the intervention gave them the opportunity to stay independent [14,18]. The initiatives also provided an outlet to address issues older adults did not want to face due to fear of losing their independence or health [14]. One study specifically implemented empowerment strategies, designed to enhance independence and self-efficacy, and saw an 84% engagement rate [18]. Participants of an arts-based activity program remarked that the initiative made them feel like contributing members of the senior’s center and their greater community [28]. Overall, older adults stated that the health promotion initiatives inspired confidence in their current and future abilities through the building of awareness and tools to address their health [16,20].

Gender differences: Gender differences in participation were apparent in the literature reviewed (n=11), with a disproportionate number of female participants included in the population samples of the studies. In seven studies, over 70% of the sample were women [15-17, 20-22,24]. While some of this discrepancy can be explained by the higher life expectancy and 60% portion of the elderly population women make up [29], several studies had included participants who were only 60 years old [16,17,21,22,24]. This makes it unlikely that participation rates would be impacted by gender differences in life expectancy. Other influencers to one’s participation, including social beliefs that men ought to “handle their own health,” may potentially act as a barrier to accessing health promotion initiatives [29].

Discussion

The objective of this paper was to summarize the empirical literature on health promotion initiatives for community-dwelling older adults in Canada. Additionally, this paper sought to describe existing programs to provide recommendations for future planning and implementation of health promotion interventions and policy for older adults. This review demonstrated that health promotion initiatives offer older adults living in the community a chance to age successfully by providing social engagement and empowerment in late life [4]. Overall, the desire of older Canadians to participate in these initiatives was substantial, displaying aspirations to learn, gain control over their health, and increase connection with the community [12,16,24]. Unfortunately, the availability of preventative services is limited and unevenly distributed across the country, mostly benefiting those in densely populated provinces and cities. Therefore, it is recommended to increase funding and prioritize primary prevention initiatives for older adults given the need and desire for such services.

This review demonstrated that programs that combined programming tools and/or utilized multi-disciplinary approaches show the most promise. For instance, combined exercise and education programs reported positive results in many biopsychosocial aspects [20,23,24], in comparison to statistically insignificant effects of these facilitation methods used separately [13,19]. Although this review is not conclusive in determining best practices for program facilitation for older adults, it suggests that multifactorial programming may work better to address the complex needs of older adults. More research into effective methods for behavior change for this demographic is needed to determine best practices for program delivery.

The reviewed literature also showed considerable diversity in health promotion programs’ facilitators. Leveraging the capacity of allied health professionals and local volunteers in community settings seemed to be a successful and efficient use of health human resources. Given that availability of resources can become a contention point in the delivery of public health programming, this speaks to the opportunities to rely on alternative facilitators for health promotion implementation.

The overall diversity of health promotion programming shown in this review demonstrates the versatility of health promotion for addressing various health needs of older adults. Especially during the current coronavirus disease 2019 (COVID-19) pandemic, these initiatives could promote positive mental health during a time when older persons are at the greatest health risk. While older adults may struggle with accessing technological devices for remote health promotion programming, Botner’s initiative can be modeled to improve technical literacy and increase social connectedness. Providing support to older adults willing to navigate technology can enable them to stay connected to their community, serving as a protective factor for adverse health outcomes [12].

The reviewed studies brought to light the gendered nature of the participation of older adults in health promotion programs. Although women make up 60% of the elderly population and have a longer life expectancy [29], several studies included in this review had quite a young sample that would likely not be impacted by these differences. While cultural and structural barriers might be a reason for poor uptake of these programs among male participants, given their overall poorer health outcomes, it is imperative to consider how their participation in these types of initiatives can be increased [29].

Additionally, the findings from this review demonstrate the ability of health promotion to increase successful aging among older adults [4]. The programs addressed all three aspects of successful aging, including a focus on improving health (physical and/or mental) and influencing active engagement in life. While most reviewed studies aimed to address physical health, an increased sense of independence reported by older adults was also significantly present. Offering tools to empower older adults to take control over their lives demonstrated positive effects on self-efficacy, which has been considered a primary indicator for the prevention of adverse health outcomes, like social isolation, in older individuals [12]. In a period of life where independence and confidence can diminish, utilizing the positive effects of health promotion to maintain self-efficacy is crucial.

Implications

The paucity of research on primary prevention initiatives for older adults in Canada suggests that the literature continues to be dominated by studies focused on disease management interventions. This not only perpetuates the ageist idea that illness and decline in old age is inevitable, but also neglects to acknowledge broader social determinants that contribute to 75% of influences on health [30]. Therefore, given the increasing number of healthy, community-dwelling older adults who continue to thrive into late life, a shift is needed in how older adults’ health needs are approached. Expansion of knowledge, both through research and program investment, on primary prevention and the many determinants that affect older persons’ health would effectively fill this gap and further promote empowered aging. The small body of literature analyzed in this review demonstrates the positive outcomes had on older adults when the investment into preventive initiatives is made. Although this study examined the literature in the Canadian context, population aging is a universal phenomenon, and these findings may be applicable to other countries where healthy aging policy is identified as a key priority.

Overall, many recommendations for future health promotion program and policy implementation can be drawn from the findings of this review. In addition to increasing availability, the implementation of interventions should be evenly dispersed across the country to allow for equal access. Also, multifactorial facilitation methods may reap the most health benefits in programming for this population. Additionally, due to the gendered nature of older adults’ participation in the health promotion programs included in this review, greater attention to potential barriers should be considered when program planning. Lastly, the versatility of health promotion for older adults demonstrates its capability to address a variety of concerns in late life. Therefore, these interventions should be used in greater capacities to address current public health issues and the social determinants of health, such as digital connectedness and the barriers to technological literacy in the age of COVID-19.

Limitations

This study has some limitations. The search was limited to empirical evidence published in the last 20 years, written in English, and based in Canada. It is possible that key studies published beyond this timeframe, in a different language, or outside of Canada were not consulted for this review. Moreover, while the focus of this review was on evidence-based health promotion interventions, there is a potential that due to the exclusion of gray literature from the review, some promising programs were missed. Finally, consistent with the chosen scoping review methodology, this review focused on a summary of the published literature and did not critically appraise the quality or strength of findings [11]. Notwithstanding these limitations, this scoping review is a first step in summarizing the availability and potential of health promotion programs for community-dwelling older adults in the Canadian context, with the ability to translate these learnings to aging populations in other countries of similar demographics.

## Conclusions

This scoping review demonstrates a wide variety of programs implemented across Canada, focused on primary prevention and health promotion for community-dwelling older adults. Recommendations can be drawn from the literature to increase the availability of health promotion initiatives for older adults across the country, utilize multifactorial programming methods, harness the ability of diverse program facilitators, expand geriatric interventions to go beyond physical health (and focus on crucial factors like the social determinants of health), and work towards addressing older men’s barriers of participation. The results show that there is promise in investing in health promotion and preventative services for the aging population, with the need for further investigation into the expansion of these services to ensure the changing needs of increasingly healthier and independent older adults are met.
